# Supplementary material for: Complexome profiling of the Chlamydomonas psb28 mutant reveals TEF5 as an early PSII assembly factor
Source: Plant Cell. 2025 Mar 17;37(6):koaf055. doi: 10.1093/plcell/koaf055 (PMC12164590; doi:10.1093/plcell/koaf055)
Supplement: koaf055_Supplementary_Data [file koaf055_supplementary_data.zip › psb28_interactive.zip!/plots/intensity/qualityPlots/peptides/EGY1_Cre03.g206929.t1.1.html]

### Cre03.g206929.t1.1

Trivial name: EGY1  
SeqCoverage [median]: 12.20  
Mass: 63.45 kDa  
  
 

| Sequence | MissedCleavage | Mass [Da] | wtIntensity | muIntensity | Score |
| --- | --- | --- | --- | --- | --- |
| AATFGPR | 0 | 718.38 | 1380.08 | 3222.16 | 103.02 |
| ASTEENQEQR | 0 | 1190.52 | 0.00 | 496.87 | 131.22 |
| DNVSPVSDAK | 0 | 1030.49 | 0.00 | 1088.12 | 93.60 |
| DNVSPVSDAKR | 1 | 1186.59 | 0.00 | 479.32 | 49.63 |
| MMQAAYGR | 0 | 926.41 | 1518.15 | 2995.71 | 143.02 |
| QLADAQR | 0 | 800.41 | 3410.60 | 4660.57 | 134.66 |
| SATAVAESVSSDAAR | 0 | 1420.68 | 7194.76 | 21135.62 | 203.51 |
| SGNVEAQQQQLR | 0 | 1356.67 | 0.00 | 2100.62 | 112.71 |
| YIKDNVSPVSDAK | 1 | 1434.74 | 395.37 | 558.44 | 73.47 |
| YIKDNVSPVSDAKR | 2 | 1590.84 | 0.00 | 476.67 | 64.24 |
|  |  |  | 13898.9 | 37214.1 |  |
